# Manipulation of Host Diet To Reduce Gastrointestinal Colonization by the Opportunistic Pathogen *Candida albicans*

**DOI:** 10.1128/mSphere.00020-15

**Published:** 2015-11-18

**Authors:** Kearney T. W. Gunsalus, Stephanie N. Tornberg-Belanger, Nirupa R. Matthan, Alice H. Lichtenstein, Carol A. Kumamoto

**Affiliations:** a Training in Education and Critical Research Skills (TEACRS) Program, Tufts University, Boston, Massachusetts, USA; b Department of Molecular Biology and Microbiology, Tufts University School of Medicine, Boston, Massachusetts, USA; c Jean Mayer USDA Human Nutrition Research Center, Cardiovascular Nutrition Laboratory, Tufts University, Boston, Massachusetts, USA; Carnegie Mellon University

**Keywords:** microbiome, commensal, pathogenesis, carbon metabolism, *Candida*, *Candida albicans*, host-pathogen interactions, medium-chain fatty acids, fatty acids

## Abstract

*Candida albicans*, the most common human fungal pathogen, can cause systemic infections with a mortality rate of ~40%. Infections arise from colonization of the gastrointestinal (GI) tract, where *C. albicans* is part of the normal microflora. Reducing colonization in at-risk patients using antifungal drugs prevents *C. albicans*-associated mortalities. *C. albicans* provides a clinically relevant system for studying the relationship between diet and the microbiota as it relates to commensalism and pathogenicity. As a first step toward a dietary intervention to reduce *C. albicans* GI colonization, we investigated the impact of dietary lipids on murine colonization by *C. albicans*. Coconut oil and its constituent fatty acids have antifungal activity *in vitro*; we hypothesized that dietary coconut oil would reduce GI colonization by *C. albicans*. Colonization was lower in mice fed a coconut oil-rich diet than in mice fed diets rich in beef tallow or soybean oil. Switching beef tallow-fed mice to a coconut oil diet reduced preexisting colonization. Coconut oil reduced colonization even when the diet also contained beef tallow. Dietary coconut oil also altered the metabolic program of colonizing *C. albicans* cells. Long-chain fatty acids were less abundant in the cecal contents of coconut oil-fed mice than in the cecal contents of beef tallow-fed mice; the expression of genes involved in fatty acid utilization was lower in *C. albicans* from coconut oil-fed mice than in *C. albicans* from beef tallow-fed mice. Extrapolating to humans, these findings suggest that coconut oil could become the first dietary intervention to reduce *C. albicans* GI colonization.

**IMPORTANCE** *Candida albicans*, the most common human fungal pathogen, can cause infections with a mortality rate of ~40%. *C. albicans* is part of the normal gut flora, but when a patient’s immune system is compromised, it can leave the gut and cause infections. By reducing the amount of *C. albicans* in the gut of susceptible patients, infections (and the resulting fatalities) can be prevented. Currently, this is done using antimicrobial drugs; to “preserve” drugs for treating infections, we looked for a dietary change to reduce the amount of *C. albicans* in the gut. Using a mouse model, we showed that adding coconut oil to the diet could become the first drug-free way to reduce *C. albicans* in the gut. More broadly, this model lets us study the interactions between our diet and the microbes in our body and the reasons why some of those microbes, under certain conditions, cause disease.

**Podcast**: A podcast concerning this article is available.

## Abstract (precis)

*Candida albicans*, the most common human fungal pathogen, can cause infections with a mortality rate of ~40%. *C. albicans* is part of the normal gut flora, but when a patient’s immune system is compromised, it can leave the gut and cause infections. By reducing the amount of *C. albicans* in the gut of susceptible patients, infections (and the resulting fatalities) can be prevented. Currently, this is done using antimicrobial drugs; to “preserve” drugs for treating infections, we looked for a dietary change to reduce the amount of *C. albicans* in the gut. Using a mouse model, we showed that adding coconut oil to the diet could become the first drug-free way to reduce *C. albicans* in the gut. More broadly, this model lets us study the interactions between our diet and the microbes in our body and the reasons why some of those microbes, under certain conditions, cause disease.

## INTRODUCTION

*Candida albicans*, a member of the endogenous human microflora, is the most common human fungal pathogen. While in most healthy individuals *C. albicans* is a harmless commensal colonizing the skin and gastrointestinal (GI) tract, when its growth advances unchecked, *C. albicans* can cause superficial mucosal candidiasis, such as oral thrush and vaginal yeast infections. Particularly in immunocompromised patients, *C. albicans* can enter the bloodstream and cause invasive or disseminated candidiasis, affecting internal organs such as the kidneys, liver, spleen, lungs, brain, and heart valves. Disseminated candidiasis is difficult to diagnose and treat; although estimates of attributable mortality vary greatly, in a large case-control study, Gudlaugsson et al. reported a candidemia-attributable mortality rate of 49% (1). It has thus been proposed that the most effective way to reduce candidemia-associated mortalities is to prevent infections from occurring (2).

Current evidence suggests that *C. albicans* infections most often arise from colonization of the patient’s own gastrointestinal tract; colonization can then spread to multiple sites in the body, which is an independent risk factor for the development of systemic infection (3). The incidence of invasive disease can therefore be reduced by decreasing colonization in patients at risk of developing infections by using antifungal prophylaxis; this has been shown to reduce mucosal and invasive candidiasis (4–14) and *Candida*-associated mortalities (4, 15–18). However, the use of antifungal drugs leads to the emergence of antifungal-resistant strains (19–22). Given that the Centers for Disease Control and Prevention have classified fluconazole-resistant *Candida* as a “serious threat” (23), it is clear that alternative methods of reducing *C. albicans* colonization are needed. It is well established that changes in diet, such as switching to a high-fat diet, can alter the gastrointestinal microflora (24–28; reviewed in references 29 and 30), but the effects of diet on *C. albicans* colonization have not been extensively studied.

**FIG 1:**
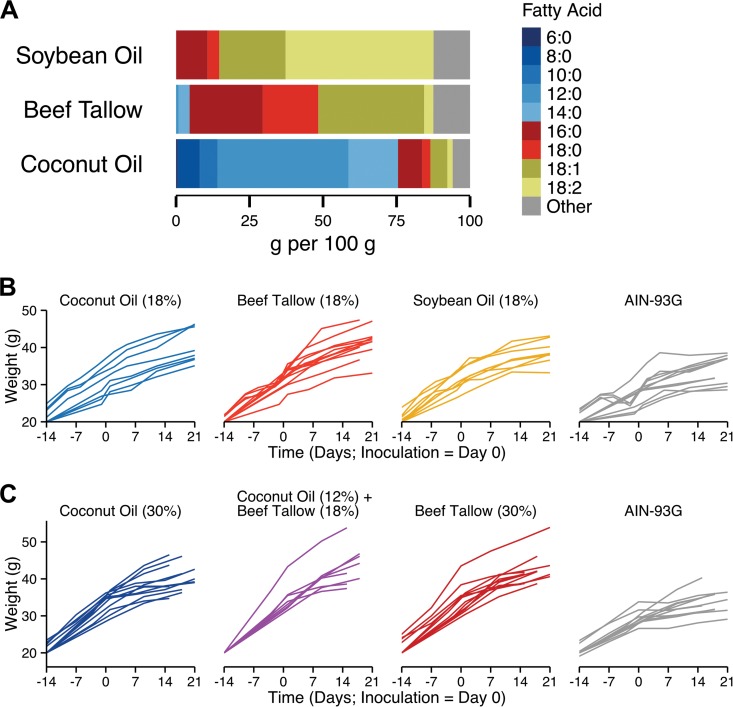
Fatty acid composition of beef tallow, soybean oil, and coconut oil and weight of mice fed diets containing those fats. (A) Fatty acid composition data from the USDA Nutritional Nutrient Database for Standard Reference (release 27, http://ndb.nal.usda.gov/). (B) Mice fed a high-fat diet containing coconut oil, beef tallow, or soybean oil (18% by weight) or a standard diet (AIN-93G) were inoculated with *C. albicans* by oral gavage on day 0 (*n* = 8 to 12 mice per diet; mice from Fig. 2). Mice were weighed periodically throughout the experiment and were sacrificed 21 days postinoculation. (C) Mice fed a high-fat diet containing either coconut oil or beef tallow (30% by weight), a high-fat diet containing both (12% coconut oil and 18% beef tallow), or a standard diet (AIN-93G) were inoculated with *C. albicans* by oral gavage on day 0 (*n* = 8 to 12 mice per diet; mice from Fig. 4). Mice were weighed periodically throughout the experiment and were sacrificed 21 days postinoculation.

The goal of this study was to test the effect of a dietary intervention on GI colonization with *C. albicans*. Coconut oil is a natural product that has been extensively studied; its chemical composition is known (see Fig. 1), and in addition to its long history of use as a dietary fat, there are decades of research showing that coconut oil and the fatty acids that it contains are safe and well tolerated when ingested or applied topically, in both animals and humans. Coconut oil and its constituent fatty acids, particularly decanoic (10:0) and dodecanoic (12:0) acids, have been shown to both inhibit the growth of and kill *C. albicans* *in vitro* (31–33). In contrast, *C. albicans* can grow using long-chain fatty acids (LCFAs), such as those found in beef tallow and soybean oil, as a sole carbon source; for instance, the growth of *C. albicans* on oleate (18:1) has been extensively studied (34–38; reviewed in reference 39). We hypothesized that dietary coconut oil would reduce GI colonization by *C. albicans in vivo*. We therefore compared the effects of dietary beef tallow, soybean oil, and coconut oil on *C. albicans* colonization in a murine model.

## RESULTS

### *C. albicans* GI colonization is lower in coconut oil-fed mice than in beef tallow- or soybean oil-fed mice.

To assess the relative effects of different sources of dietary fat on gastrointestinal (GI) colonization by *C. albicans*, we compared the effects of dietary beef tallow, soybean oil, and coconut oil on *C. albicans* colonization in a murine model. Beef tallow and soybean oil are rich in long-chain saturated fatty acids (16:0 and 18:0) and unsaturated fatty acids (18:1 and 18:2), respectively. In contrast, coconut oil is rich in medium-chain fatty acids (MCFAs; 8:0, 10:0, and 12:0) (Fig. 1A). We hypothesized that dietary coconut oil would reduce GI colonization by *C. albicans*.

**FIG 2:**
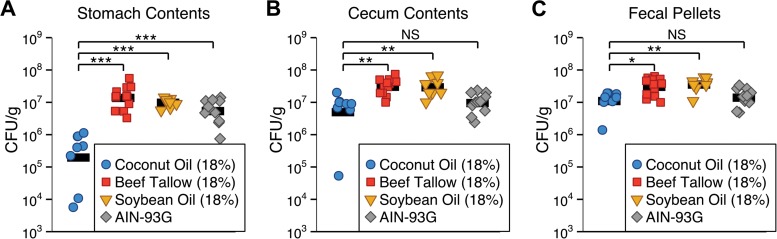
*C. albicans* murine gastrointestinal colonization is lower in mice fed a diet containing coconut oil than in mice fed a diet containing beef tallow or soybean oil. Mice fed a high-fat diet containing coconut oil, beef tallow, or soybean oil (18% by weight) or a standard diet (AIN-93G) were inoculated with *C. albicans*, and colonization (CFU per gram of material) was determined 21 days postinoculation. (A) Colonization was significantly lower in the stomach contents of mice fed the coconut oil diet than in the stomach contents of mice fed the beef tallow diet, soybean oil diet, or AIN-93G. (B) Colonization was significantly lower in the cecal contents of mice fed the coconut oil diet than in the cecal contents of mice fed the beef tallow or soybean oil diet but not AIN-93G. (C) Colonization was significantly lower in the fecal pellets of mice fed the coconut oil diet than in the fecal pellets of mice fed the beef tallow or soybean oil diet but was not significantly different from colonization in mice fed AIN-93G. Each symbol represents one mouse (*n* = 8 to 12 mice per diet); bars represent geometric means. NS, *P* > 0.05; *, *P* < 0.05; **, *P* < 0.01; ***, *P* < 0.001, Tukey’s HSD test.

Mice fed either a high-fat diet containing either coconut oil, beef tallow, or soybean oil or a standard diet (AIN-93G) were orally inoculated with *C. albicans*, and gastrointestinal colonization was measured 21 days postinoculation. Colonization was significantly lower in the stomach contents of mice fed the coconut oil diet than in the stomach contents of mice fed the beef tallow diet (*P* < 0.0001), soybean oil diet (*P* < 0.0001), or AIN-93G (*P* < 0.0001) (Fig. 2). Similarly, colonization was significantly lower in the cecal contents of mice fed the coconut oil diet than in the cecal contents of mice fed the beef tallow diet (*P* = 0.002) or soybean oil diet (*P* = 0.007) (Fig. 2B). Colonization was significantly lower in the fecal pellets of mice fed the coconut oil diet than in the fecal pellets of mice fed the beef tallow diet (*P* = 0.01) or soybean oil diet (*P* = 0.007) (Fig. 2C). No significant difference in colonization between beef tallow- and soybean oil-fed mice was observed (*P* > 0.9). Hence, for the remainder of the experiments we focused on the comparison between dietary coconut oil and beef tallow.

### Changing to a coconut oil-containing diet reduces preexisting GI colonization by *C. albicans.*

**FIG 3:**
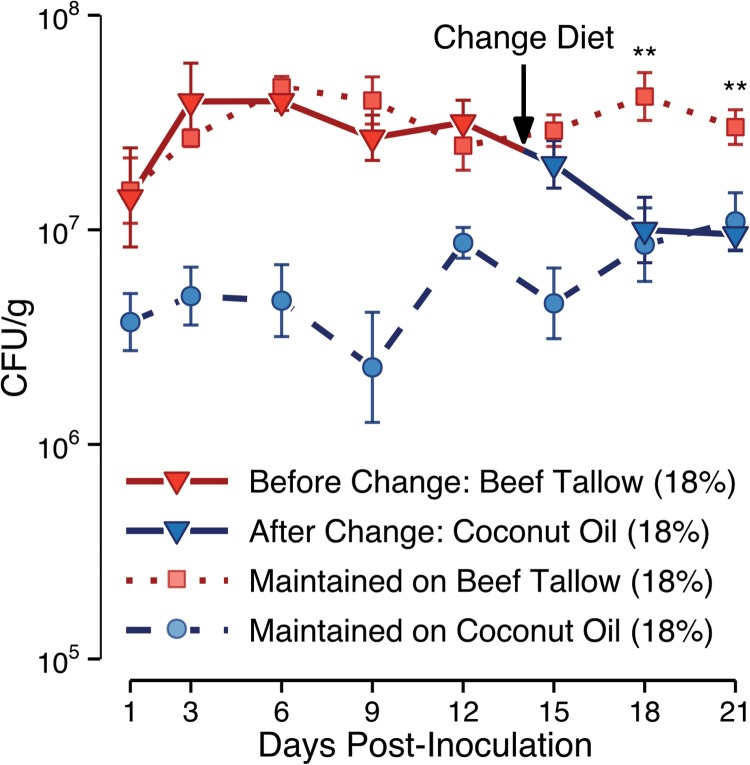
Changing to a coconut oil-containing diet reduces preexisting GI colonization by *C. albicans*. Mice on a beef tallow-containing diet (18% by weight) were inoculated with *C. albicans*, and colonization was measured using fecal pellets collected on the days indicated. Fourteen days postinoculation, mice were switched to a coconut oil-containing diet (18% by weight); data from mice maintained on the beef tallow- or coconut oil-containing diet throughout the experiment are shown for comparison. Eighteen days postinoculation (4 days after the change in diet), colonization in mice switched from the beef tallow to the coconut oil diet was lower than that in mice maintained on the beef tallow diet and was not significantly different from that in mice fed the coconut oil-containing diet throughout the experiment. Data shown as geometric means ± standard errors; *n* = 8 to 12 mice per diet. **, *P* ≤ 0.01, Tukey’s HSD test.

To determine whether dietary coconut oil affects colonization by preventing *C. albicans* from establishing robust GI colonization, or whether it could reduce preexisting colonization, a crossover diet experiment was performed. Mice inoculated with *C. albicans* were maintained on the beef tallow diet for 14 days postinoculation to establish robust GI colonization, as monitored using fecal pellets (Fig. 3**,** <14 days). The mice were then switched to the coconut oil diet for 7 days. When mice were switched from the beef tallow diet to the coconut oil diet, *C. albicans* GI colonization decreased; 4 days after the change in diet, colonization was as low in the mice switched from the beef tallow diet to the coconut oil diet as in the mice fed the coconut oil diet throughout the experiment (*P* = 0.9) and was lower than colonization in mice fed the beef tallow diet throughout the experiment (*P* = 0.01) (Fig. 3). These data demonstrate that a change in diet can reduce preexisting GI colonization by *C. albicans*.

### Dietary coconut oil inhibits GI colonization by *C. albicans.*

**FIG 4:**
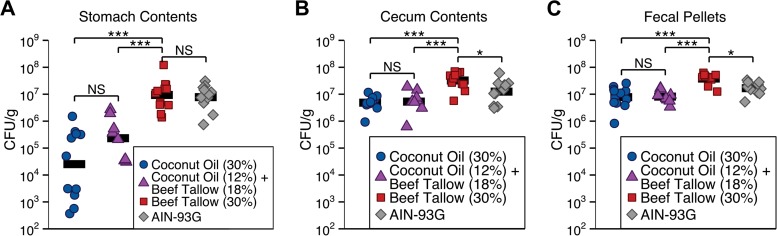
Dietary coconut oil inhibits GI colonization by *C. albicans*. Mice fed a high-fat diet containing both coconut oil (12% by weight) and beef tallow (18%) or isocaloric diets containing either coconut oil or beef tallow (30%) or a standard diet (AIN-93G) were inoculated with *C. albicans*, and CFU per gram of material was determined 21 days postinoculation. (A) Colonization was significantly lower in the stomach contents of mice fed the coconut oil diet or the diet containing both coconut oil and beef tallow than in the stomach contents of mice fed the beef tallow diet. (B) Colonization was significantly lower in the cecal contents of mice fed the coconut oil diet, the diet containing both coconut oil and beef tallow, or AIN-93G than in the cecal contents of mice fed the beef tallow diet. (C) Colonization was significantly lower in the fecal pellets of mice fed the coconut oil diet, the diet containing both coconut oil and beef tallow, or AIN-93G than in the fecal pellets of mice fed the beef tallow diet. Each symbol represents one mouse (*n* = 8 to 12 mice per diet); bars represent geometric means. NS, *P* > 0.05; *, *P* < 0.05; **, *P* < 0.01; ***, *P* ≤ 0.001, Tukey’s HSD test.

There are two types of mechanisms by which coconut oil could reduce GI colonization by *C. albicans*: coconut oil could lack factors required for robust GI colonization or could actively inhibit colonization (such as by killing *C. albicans* [31]). If coconut oil alone is insufficient to support robust colonization, then this colonization defect should be rescued by the addition of beef tallow. Alternatively, if coconut oil actively inhibits colonization, it may do so in the presence of beef tallow. To distinguish between these possibilities, colonization was measured in mice fed a high-fat diet containing either coconut oil or beef tallow, a high-fat diet containing both coconut oil and beef tallow, or a standard diet (AIN-93G). Colonization was significantly lower in the stomachs, ceca, and fecal pellets of mice fed the coconut oil diet (*P* < 0.0001) or the diet containing both coconut oil and beef tallow (*P* < 0.001) than in those of mice fed the beef tallow-only diet (Fig. 4). No significant difference in colonization was observed between mice fed the diet containing both coconut oil and beef tallow and mice fed the coconut oil-only diet (stomach contents, *P* = 0.08; cecal contents and fecal pellets, *P* > 0.9). Coconut oil, therefore, reduced colonization even in the presence of beef tallow, suggesting that coconut oil actively inhibits GI colonization by *C. albicans*.

### Dietary coconut oil alters the fatty acid composition of the GI contents.

We hypothesized that coconut oil might inhibit *C. albicans* colonization by altering the fatty acids present in the GI environment. To determine how different dietary fats might impact *C. albicans* colonization, we therefore began by investigating the fatty acid composition of the GI contents.

**FIG 5:**
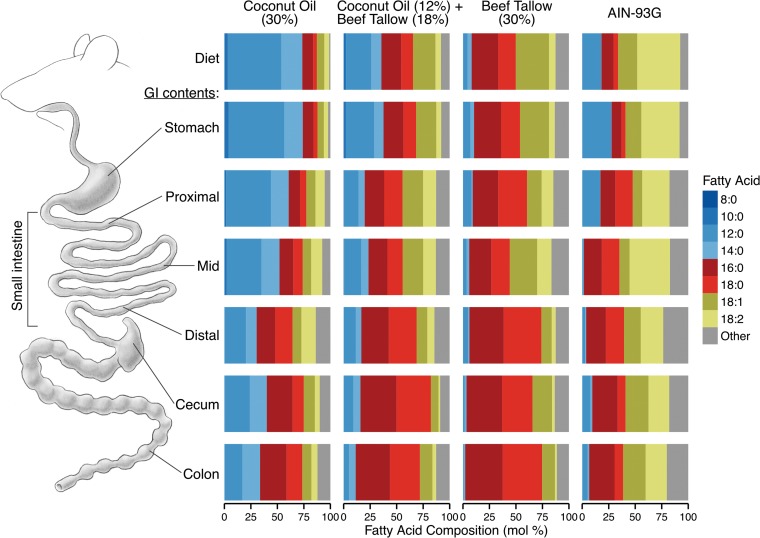
Fatty acid profile (molar percentage) of the GI contents of mice fed diets containing either coconut oil or beef tallow, a diet containing both coconut oil and beef tallow, or a standard diet (AIN-93G). To determine the effect of dietary coconut oil and beef tallow on the fatty acid composition of the GI contents, mice were fed diets containing coconut oil or beef tallow (30%), a diet containing both (12% coconut oil and 18% beef tallow), or AIN-93G. Organ contents were harvested from throughout the GI tract, and the fatty acid profiles of these samples, as well as of the original diets, were determined by gas chromatography and expressed as molar percentages. Data represent the averages for three mice per diet.

**FIG 6:**
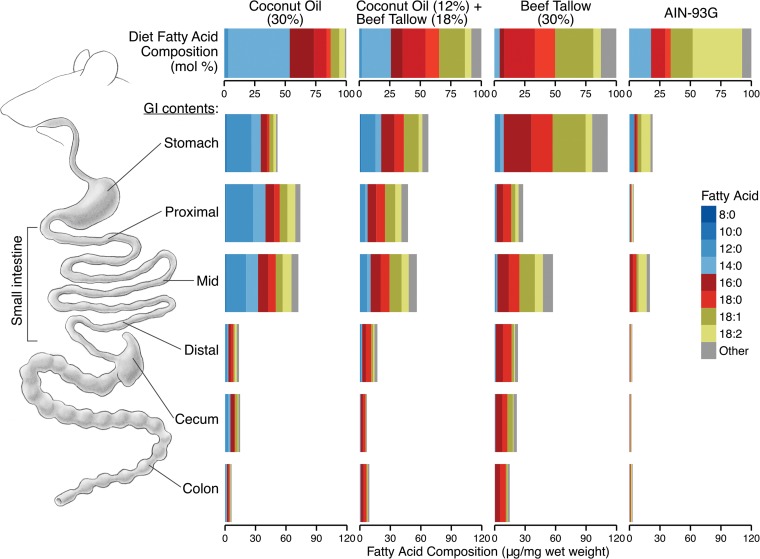
Fatty acid profile (micrograms per milligram, wet weight) of the GI contents of mice fed diets containing either coconut oil or beef tallow, a diet containing both coconut oil and beef tallow, or a standard diet (AIN-93G). To determine the effect of dietary coconut oil and beef tallow on the fatty acid composition of the GI contents, mice were fed diets containing coconut oil or beef tallow (30%), a diet containing both (12% coconut oil and 18% beef tallow), or AIN-93G. Organ contents were harvested from throughout the GI tract, and the fatty acid profiles of these samples, as well as of the original diets, were determined by gas chromatography and expressed as micrograms of fatty acid per milligram (wet weight). Data represent the averages for three mice per diet.

As expected, the fatty acid composition of the GI contents (as a molar percentage of total fatty acids) reflected the fatty acid composition of the original diets. Both the beef tallow diet and the GI contents of beef tallow-fed mice contained predominantly long-chain fatty acids (LCFAs), particularly hexadecanoic (16:0) and octadecanoic (18:0) acids. In contrast, the coconut oil diet and the GI contents of coconut oil-fed mice were rich in medium-chain fatty acids (MCFAs), especially decanoic (10:0) and dodecanoic (12:0) acids (Fig. 5). Consistent with previous reports describing the absorption of MCFAs in the small intestine, the abundance of MCFAs (micrograms of fatty acid per milligram of GI contents) decreased between the stomach and the distal small intestine (Fig. 6).

**FIG 7:**
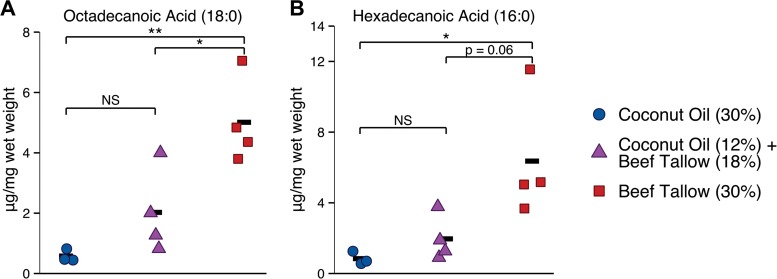
Long-chain fatty acids are less abundant in the cecal contents of coconut oil-fed mice than in the cecal contents of beef tallow-fed mice. Mice were fed a high-fat diet containing either coconut oil or beef tallow (30% by weight) or both (12% coconut oil and 18% beef tallow). (A) The concentration of octadecanoic acid (18:0) was significantly lower in the cecal contents of mice fed the coconut oil diet or the diet containing both coconut oil and beef tallow than in the cecal contents of mice fed the beef tallow diet. (B) The concentration of hexadecanoic acid (16:0) was significantly lower in the cecal contents of mice fed the coconut oil diet than in the cecal contents of mice fed the beef tallow diet; the concentration of hexadecanoic acid was also lower in the cecal contents of mice fed the diet containing both coconut oil and beef tallow than in the cecal contents of beef tallow-fed mice, but this difference was not statistically significant (*P* = 0.06). Composite results from two experiments are shown, one in which *n* was 3 mice per diet and one in which samples from three mice per diet were pooled. Each symbol represents one sample; bars represent averages. NS, *P* > 0.05; *, *P* < 0.05; **, *P* < 0.01, Tukey’s HSD test.

To determine whether the concentration of fatty acids in the GI tract varied significantly between diets, the fatty acids in cecal contents were measured. Significant diet-dependent differences were detected in the concentrations of the long-chain fatty acids octadecanoic acid (18:0; one-way analysis of variance [ANOVA], *F*2,8 = 12.16, *P* = 0.0038) and hexadecanoic acid (16:0; *F*2,8 = 5.9, *P* = 0.027). The concentration of octadecanoic acid was significantly lower in the cecal contents of mice fed the coconut oil diet (*P* = 0.004) or the diet containing both coconut oil and beef tallow (*P* = 0.02) than in the cecal contents of mice fed the high-colonization beef tallow diet (Fig. 7A). There was not a significant difference in the concentrations of octadecanoic acid between mice fed the coconut oil diet and mice fed the diet containing both coconut oil and beef tallow (*P* = 0.3). Similarly, the concentration of hexadecanoic acid was significantly lower in the cecal contents of mice fed the coconut oil diet (*P* = 0.03) than in the cecal contents of mice fed the beef tallow diet; the concentration of hexadecanoic acid was also lower in mice fed the diet containing both coconut oil and beef tallow than in beef tallow-fed mice, but this difference was not statistically significant (*P* = 0.06) (Fig. 7B). There was no significant difference in the concentrations of hexadecanoic acid between mice fed the coconut oil diet and mice fed the diet containing both coconut oil and beef tallow (*P* = 0.8). Medium- and longer-chain fatty acids (10:0, 12:0, 14:0, and 20:0) were present at levels near the limit of detection, and no significant differences in abundance were detected (one-way ANOVA, *P* > 0.1). This suggests that the reduced availability of long-chain fatty acids in the cecal contents of mice fed the coconut oil-containing diets may have contributed to the reduced colonization observed in coconut oil-fed mice.

### Expression of *C. albicans* fatty acid catabolic genes is lower in coconut oil-fed mice than in beef tallow-fed mice.

Unlike mammals, fungi can grow using lipids as a sole carbon source, as fungi can produce both energy and sugars (essential biosynthetic precursors) from fatty acids. Long-chain fatty acids were less abundant in the cecal contents of coconut oil-fed mice than in the cecal contents of mice fed the beef tallow diet. This suggested that there would be less fatty acid catabolism occurring in *C. albicans* colonizing the ceca of coconut oil-fed mice. To test this hypothesis, we measured the expression of *C. albicans* genes in the cecal contents of mice (from Fig. 4) fed diets containing either coconut oil or beef tallow, a diet containing both coconut oil and beef tallow, or the AIN-93G diet.

**FIG 8:**
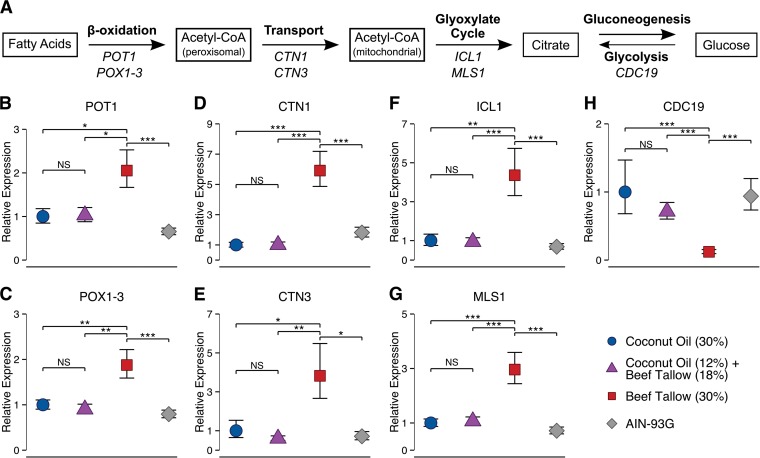
Expression of *C. albicans* fatty acid catabolic genes is lower in the cecal contents of coconut oil-fed mice than in the cecal contents of beef tallow-fed mice. (A) Schematic of fatty acid catabolism in *C. albicans*. The pathways of fatty acid β-oxidation, acetyl-CoA transport (carnitine shuttle), the glyoxylate cycle, gluconeogenesis, and glycolysis are depicted, and the names of *C. albicans* genes differentially regulated in response to experimental diets are shown. (B to H) The expression of *C. albicans* genes was measured in the cecal contents of mice fed a high-fat diet containing either coconut oil or beef tallow (30%) or both (12% coconut oil and 18% beef tallow) or a standard diet (AIN-93G). (B to G) Expression of the fatty acid β-oxidation genes *POT1* (B) and *POX1*-*3* (C), the carnitine acetyltransferase genes *CTN1* (D) and *CTN3* (E), and the glyoxylate cycle genes *ICL1* (F) and *MLS1* (G) was lower in *C. albicans* from mice fed the coconut oil diet or the diet containing both beef tallow and coconut oil than in *C. albicans* from mice fed the beef tallow diet. (H) Expression of the glycolytic gene *CDC19* was higher in *C. albicans* from mice fed the coconut oil diet or the diet containing both beef tallow and coconut oil than in *C. albicans* from mice fed the beef tallow diet. Data are expressed as fold change relative to the mean expression in mice fed the coconut oil diet; all gene expression data (measured via RT-qPCR) have been normalized to reference genes. Symbols indicate means ± standard errors; *n* = 8 to 12 mice per diet. NS, *P* > 0.05; *, *P* < 0.05; **, *P* < 0.01; ***, *P* < 0.001, Bonferroni-corrected pairwise *t* test following one-way ANOVA.

When used by *C. albicans* as a carbon or energy source, fatty acids are first broken down via fatty acid β-oxidation to produce acetyl coenzyme A (acetyl-CoA) (Fig. 8A). The expression of fatty acid β-oxidation genes was significantly lower in *C. albicans* from mice fed the coconut oil diet (*POT1*, *P* = 0.033; *POX1*-*3*, *P* = 0.008) or the diet containing both coconut oil and beef tallow (*POT1*, *P* = 0.036; *POX1*-*3*, *P* = 0.002) than in *C. albicans* from mice fed the beef tallow diet (Fig. 8B and C). No significant difference in the expression of either gene was observed between *C. albicans* from mice fed the coconut oil diet and *C. albicans* from mice fed the diet containing both coconut oil and beef tallow (*P* = 1.0). A similar trend was observed for other β-oxidation genes (data not shown). Therefore, *C. albicans* from coconut oil-fed mice may be producing less acetyl-CoA from fatty acids than *C. albicans* from beef tallow-fed mice.

In yeasts such as *C. albicans*, fatty acid β-oxidation occurs exclusively in the peroxisome (reviewed in reference 40). The resulting acetyl-CoA must then be transported to the mitochondria; however, acetyl-CoA cannot readily cross plasma membranes. Peroxisomal acetyl-CoA is therefore converted to acetyl-carnitine for transport to the mitochondria, where it is converted back into acetyl-CoA; these conversions are catalyzed by carnitine acetyltransferases (34, 35, 37; reviewed in reference 39). The expression of carnitine acetyltransferase genes was significantly lower in *C. albicans* from mice fed the coconut oil diet (*CTN1*, 5.9-fold, *P* < 0.0001; *CTN3***,** 3.8-fold, *P* = 0.05) or the diet containing both coconut oil and beef tallow (*CTN1*, 5.9-fold, *P* < 0.0001; *CTN3*, 6.3-fold, *P* = 0.0035) than in *C. albicans* from mice fed the beef tallow diet (Fig. 8D and E). No significant difference in the expression of either gene was observed between *C. albicans* from mice fed the coconut oil diet and *C. albicans* from mice fed the diet containing both coconut oil and beef tallow (*P* = 1.0). This suggests that there may be less acetyl-carnitine transport occurring in *C. albicans* from coconut oil-fed mice than in *C. albicans* from beef tallow-fed mice.

Once in the mitochondria, either acetyl-CoA can enter the tricarboxylic acid (TCA) cycle to produce ATP, or it can be used as a substrate in the glyoxylate cycle, a variant of the TCA cycle that yields no ATP but instead enables synthesis of sugars from acetyl-CoA. The expression of the glyoxylate cycle genes *ICL1* and *MLS1* was significantly lower in *C. albicans* from mice fed the coconut oil diet (*ICL1*, 4.4-fold, *P* = 0.0016; *MLS1*, 3.0-fold, *P* = 0.0003) or the diet containing both coconut oil and beef tallow (*ICL1*, 4.6-fold, *P* = 0.0010; *MLS1*, 2.8-fold, *P* = 0.0007) than in *C. albicans* from mice fed the beef tallow diet (Fig. 8F and G). No significant difference in the expression of either gene was observed between *C. albicans* from mice fed the coconut oil diet and *C. albicans* from mice fed the diet containing both coconut oil and beef tallow (*P* = 1.0). This suggests that there is less flux through the glyoxylate cycle in *C. albicans* from coconut oil-fed mice than in *C. albicans* from beef tallow-fed mice.

Glucose is an essential biosynthetic precursor; among other things, it is required for the production of ribose and thus nucleic acids. Glucose is thought to be scarce in the GI tract. However, *C. albicans* can synthesize glucose (gluconeogenesis) using products of the glyoxylate cycle. Thus, the lower expression of glyoxylate cycle genes in *C. albicans* from coconut-oil fed mice (compared to *C. albicans* from beef tallow-fed mice) predicts that the expression of gluconeogenic genes should be lower and the expression of glycolytic genes higher in *C. albicans* from coconut oil-fed mice than in *C. albicans* from beef tallow-fed mice. During glycolysis, Cdc19p converts phosphoenolpyruvate (PEP) to citrate, which can then enter the TCA cycle. As predicted, the expression of *CDC19* was significantly higher in *C. albicans* from mice fed the coconut oil diet (8.3-fold, *P* = 0.0001) or the diet containing both coconut oil and beef tallow (5.9-fold, *P* = 0.0007) than in *C. albicans* from mice fed the beef tallow diet (Fig. 8H). No significant difference in expression was observed between *C. albicans* from mice fed the coconut oil diet and *C. albicans* from mice fed the diet containing both coconut oil and beef tallow (*P* = 1.0). This suggests that glycolysis is increased, and gluconeogenesis decreased, in *C. albicans* from coconut oil-fed mice compared to those in *C. albicans* from beef tallow-fed mice.

The expression of genes involved in the catabolism of fatty acids was lower in *C. albicans* from coconut oil-fed mice than in *C. albicans* from beef tallow-fed mice. Importantly, the expression of these genes was as low in *C. albicans* from mice fed a diet containing both coconut and beef tallow as it was in *C. albicans* from mice fed a diet containing coconut oil but no beef tallow. This implies that, while *C. albicans* used long-chain fatty acids from dietary beef tallow as a carbon source, this did not occur when the diet also contained coconut oil. These findings are consistent with the hypothesis that colonization is lower in the gastrointestinal tracts of coconut oil-fed mice than in the GI tracts of beef tallow-fed mice at least in part because the long-chain fatty acids that fuel *C. albicans* growth in beef tallow-fed mice are not available in the GI tracts of coconut oil-fed mice. Perhaps most importantly, these data demonstrate that the metabolic program of colonizing cells, which is essential for the adaptation of *C. albicans* to host niches and impacts *C. albicans* pathogenicity and commensalism, can be modified by a dietary intervention.

## DISCUSSION

Our results suggest that coconut oil could become the first dietary intervention to reduce GI colonization by *C. albicans*. Dietary coconut oil both reduced *C. albicans* murine GI colonization and altered the metabolic program of the colonizing cells. These two effects of dietary coconut oil likely occur by two different mechanisms.

Colonization was lower in mice fed a coconut oil-rich diet than in mice fed diets rich in beef tallow or soybean oil (Fig. 2), showing that dietary fats affect *C. albicans* colonization. In order to have therapeutic benefit, any dietary intervention must be able to reduce preexisting *C. albicans* colonization in patients at risk of developing candidiasis; in mice, changing to a coconut oil-containing diet significantly reduced preexisting GI colonization by *C. albicans* within 4 days (Fig. 3). Further, coconut oil actively inhibited murine GI colonization even when the diet also contained beef tallow: colonization by *C. albicans* was as low in mice fed a diet containing both coconut oil and beef tallow as in mice fed a coconut oil-rich diet without beef tallow (Fig. 4). Thus, our results suggest that adding coconut oil to a patient’s existing diet could reduce GI colonization by *C. albicans*.

Dietary coconut oil may reduce GI colonization by killing or inhibiting the growth of *C. albicans* in the GI tract. Coconut oil is composed primarily of medium-chain fatty acids (MCFAs), which are fungistatic and fungicidal for *C. albicans*. Coconut oil is ~45% dodecanoic acid (12:0) (Fig. 1A****) (41), which has been shown both to inhibit *C. albicans* growth (32) and to kill *C. albicans* within 30 min (32, 33). Similar results were found with decanoic acid (10:0), which is also present in coconut oil (32, 33, 41). In the GI tract, lipids are present predominantly as triglycerides, rather than free fatty acids; however, coconut oil has also been shown to have antifungal action against *C. albicans* *in vitro* (31). Any direct antimicrobial effects exerted by the MCFAs in coconut oil probably occur in the upper part of the GI tract, because MCFAs are mostly absorbed in the small intestine and are therefore scarce in the contents of the cecum and colon. Our observation that coconut oil has a greater impact on colonization in the stomach than on colonization in the cecum or fecal pellets is consistent with this hypothesis. Thus, the antimicrobial properties of MCFAs may contribute to the reduced colonization observed in the GI tracts of coconut oil-fed mice.

In addition to directly decreasing GI colonization by *C. albicans*, dietary coconut oil altered the metabolic program of the colonizing cells. Long-chain fatty acids were less abundant in the cecal contents of mice fed coconut oil-containing diets than in the cecal contents of mice fed a diet rich in beef tallow (Fig. 5 to 7), and the expression of genes involved in fatty acid β-oxidation, acetyl unit transport, the glyoxylate cycle, and gluconeogenesis was lower in *C. albicans* from the ceca of coconut oil-fed mice than in *C. albicans* from the ceca of beef tallow-fed mice (Fig. 8). When carbohydrates are scarce, *C. albicans* can use fatty acids as a carbon source; the pathways involved in *C. albicans* fatty acid catabolism include fatty acid β-oxidation, the glyoxylate cycle, and gluconeogenesis (42–46). The increased expression of genes involved in these pathways by *C. albicans* from the cecal contents of beef tallow-fed mice is similar to the pattern of gene expression observed in *C. albicans* under other conditions (reviewed in reference 47). These conditions include exposure to neutrophils (42, 48) and internalization by macrophages (42, 44, 46); Lorenz et al. used genome-wide transcriptional analysis of *C. albicans* to demonstrate that phagocytosis by macrophages likely induces a reprogramming of metabolism to produce glucose from fatty acids via the glyoxylate cycle (49). The ability to use a variety of carbon sources is integral to the commensalism and pathogenicity of *C. albicans*. Local nutrient availability differs widely between the diverse host niches encountered by *C. albicans*, which can harmlessly colonize a variety of body sites or cause life-threatening systemic infections of the blood and internal organs. The metabolic flexibility to assimilate available carbon sources is thus of great importance to *C. albicans*. In addition to providing nutrients for cell growth, metabolic adaptation alters a plethora of other factors that impact *C. albicans* pathogenicity, such as stress resistance (including susceptibility to antifungal drugs), cell wall structure (which influences adhesion and immune recognition), and virulence factor expression (reviewed in reference 47). Our results therefore suggest that metabolic adaptations by *C. albicans* in response to the availability of long-chain fatty acids in the GI tract may contribute to the robust colonization seen in beef tallow- and soybean oil-fed mice compared to coconut oil-fed mice. Importantly, even when the diet contained beef tallow, these metabolic adaptations were completely ablated by dietary coconut oil. Thus, our results suggest that adding coconut oil to a patient’s existing diet could both reduce colonization and alter the metabolic program of colonizing *C. albicans* cells.

The effects of coconut oil on GI colonization by *C. albicans* are likely due to its constituent medium-chain fatty acids. Because MCFAs are saturated fatty acids, one concern that is often raised about the use of coconut oil as a dietary intervention is the possible health risks associated with saturated fats. However, it seems unlikely that there would be significant long-term cardiovascular effects from consuming coconut oil as a short-term prophylactic measure. Additionally, it is not clear whether consuming the fatty acids in coconut oil has the same health effects (adverse or not) as those of eating longer-chain saturated fats. Coconut oil is rich in MCFAs, which have a chain length of 8 to 12 carbon atoms (Fig. 1A); in contrast, most dietary fats contain primarily fatty acids with a chain length of 14 or more carbons (long-chain fatty acids [LCFAs]). MCFAs are smaller and more water soluble than LCFAs, and in mammals, MCFAs and LCFAs are digested and metabolized differently (reviewed in reference 50). MCFAs are absorbed more rapidly and efficiently by the intestine; unlike that of LCFAs, the absorption of MCFAs does not require pancreatic function or bile salts. Once absorbed, MCFAs enter the bloodstream and are transported to the liver via the hepatic portal vein, whereas LCFAs are transported via the lymph system. Once in the liver, MCFAs are also metabolized differently. These physiological differences suggest that long-term consumption of MCFAs may not have the same effect on cardiovascular health as consumption of LCFAs. Thus, the efficacy of a long-term or intermittent coconut oil-based dietary intervention should be investigated as a possible treatment option for patients with chronic health conditions requiring long-term antifungal prophylaxis.

The next step toward a dietary intervention will be to determine whether the findings reported in this study can be replicated in humans at a reasonable dose. In mice, coconut oil effectively reduced colonization across a range of doses (12 to 30%); future research will be required to determine the minimum effective dose. One limitation of this study is the high fat contents of the experimental diets: the diets containing 18% and 30% fat (by weight) provide 41% and 57% of calories from fat, respectively, compared to 33% of calories from fat for the typical American diet (51). However, the diet containing both coconut oil and beef tallow, which resulted in colonization as low as that seen in mice eating the 30% coconut oil diet, provided only 22% of calories from coconut oil—the equivalent of about 3.5 tbs of coconut oil per day for a 2,000-cal diet. This suggests that a decrease in *C. albicans* colonization might be achievable by dietary supplementation with a feasible dose of coconut oil.

This is not the first attempt to identify a dietary intervention to decrease GI colonization by *C. albicans*. Previous studies have examined the effects of dietary carbohydrates on *C. albicans* colonization. Consumption of a high-glucose diet increased gastrointestinal colonization by *C. albicans* in a neutropenic mouse model (52). However, in healthy human subjects, Weig et al. found no correlation between subjects’ normal dietary carbohydrate intake and *C. albicans* GI colonization (53). Moreover, they showed that doubling the daily carbohydrate intake of their subjects did not significantly increase gastrointestinal colonization, although they detected an increase in GI colonization in a selected subset of individuals with elevated basal levels of oral *C. albicans* colonization. Our results are consistent with the conclusion that dietary carbohydrates have a minimal effect on colonization. If a reduction in dietary carbohydrates decreased *C. albicans* colonization, we would expect to see lower colonization in mice fed the high-fat diets, which are lower in carbohydrates, than in mice fed AIN-93G. However, we observed the opposite: colonization was higher in mice fed the beef tallow- and soybean oil-rich diets than in mice fed AIN-93G. Therefore, there is some evidence that a high-carbohydrate diet can increase *C. albicans* gastrointestinal colonization under certain conditions; however, there is currently no evidence that a reduction in dietary carbohydrates decreases *C. albicans* colonization of the gastrointestinal tract. Our results suggest that consumption of coconut oil may become the first dietary intervention to reduce *C. albicans* GI colonization.

In summary, our results indicate that coconut oil is an effective dietary intervention to reduce murine GI colonization by *C. albicans*. Coconut oil both decreased GI colonization by *C. albicans* and altered the metabolic profile of the colonizing cells. Our findings suggest that adding coconut oil to the diet of patients at high risk of developing invasive candidiasis might decrease *C. albicans* GI colonization and thus disease risk.

## MATERIALS AND METHODS

### Diets.

Sterilized, pelleted diets were obtained from Bio-Serv. All diets were based on the AIN-93G diet (54), which was used as the standard diet. AIN-93G contains 7% soybean oil (70 g/kg). Fat-supplemented diets contained 2% soybean oil (20 g/kg) to provide essential fatty acids and additional fat (coconut oil, beef tallow, or soybean oil) at either 18% or 30% (180 or 300 g/kg, respectively). The diet containing both beef tallow (18%) and coconut oil (12%) was compared to isocaloric diets containing either coconut oil or beef tallow (30%). The composition of all diets is presented in Table 1.

**TABLE 1:** Diet recipes

Ingredient	Concn (g/kg) in diet:						
	AIN-93G	Coconut oil (18%)	Beef tallow (18%)	Soybean oil (18%)	Coconut oil (30%)	Coconut oil (12%) plus beef tallow (18%)	Beef tallow (30%)
Cornstarch	397	208	208	208	40	40	40
Casein	200	234	234	234	266	266	266
Maltodextrin	132	141	141	141	141	141	141
Sucrose	100	100	100	100	100	100	100
Soybean oil	70	20	20	200	20	20	20
Beef tallow	0	0	180	0	0	180	300
Coconut oil	0	180	0	0	300	120	0
Fiber	50	58.4	58.4	58.4	66	66	66
Mineral mix	35	41.2	41.2	41.2	46.2	46.2	46.2
Vitamin mix	10	11.7	11.7	11.7	13.3	13.3	13.3
l-Cysteine	3	3.5	3.5	3.5	3.95	3.95	3.95
Choline bitartrate	2.5	2.9	2.9	2.9	3.3	3.3	3.3
*tert*-Butyl-hydroquinone	0.014	0.015	0.015	0.015	0.018	0.018	0.018
Dye	0.01	0.1	0.1	0.1	0.1	0.1	0.1

### Determination of gastrointestinal colonization.

All mouse protocols were approved by Tufts University’s Institutional Animal Care and Use Committee. Female Swiss Webster mice (18 to 20 g; Charles River Laboratories, Inc., Wilmington, MA; *n* = 8 to 12 mice per diet) were fed the indicated diets for 14 days prior to and 21 days following inoculation with *C. albicans*. Mice were treated with tetracycline (1 g/liter), streptomycin (2 g/liter), and gentamicin (0.1 g/liter) in their drinking water throughout the experiment beginning 4 days prior to inoculation. Mice were weighed periodically and gained weight on all diets (Fig. 1B and C). *C. albicans* laboratory strain DAY185 (kind gift of A. Mitchell, Carnegie Mellon University), derived from the well-characterized clinical isolate SC5314, was used throughout. DAY185 was grown for 24 h at 37°C in YPD (1% yeast extract, 2% peptone, 2% glucose) liquid medium, washed twice with phosphate-buffered saline (PBS), and adjusted to 5 × 10^8^ cells/ml in PBS. Mice were inoculated with *C. albicans* by oral gavage (5 × 10^7^ cells in 0.1 ml), as described previously (55). Colonization (CFU per gram of material) was monitored by collecting fecal pellets (produced within 10 min prior to collection) at various days postinoculation, homogenizing them in PBS, and plating homogenates on YPD agar medium supplemented with 50 µg/ml ampicillin and 100 µg/ml streptomycin; we have previously shown that most *C. albicans* cells in the gut are yeast, not hyphae (55). Mice were sacrificed on day 21 postinoculation, and *C. albicans* CFU per gram of material was determined in stomach and cecal contents; cecal contents were also harvested for determination of *C. albicans* gene expression by reverse-transcription quantitative PCR (RT-qPCR) as described below. Homogenates of kidneys, liver, and tongue were also plated; no colonies were observed from homogenates of these organs. Composite results from at least two experiments are shown. Colonization data were analyzed using R (56) and the R packages *nlme* (57) and *multcomp* (58). A one-way ANOVA was used to test for differences in colonization between diets at day 21 postinoculation. When colonization differed significantly between diets (*P* < 0.05), *post hoc* pairwise comparisons were performed using Tukey’s honestly significant difference (HSD) test. To look at colonization over time, a linear mixed-effects analysis was performed on the log-transformed fecal pellet data, using subjects (mice) as random effects and an autocorrelation structure of order 1 (AR1). Fixed effects included diet and day postinoculation and the interaction of diet and day. We checked for normality and homogeneity by visual inspections of plots of residuals against fitted values. To assess the validity of the mixed-effects analyses, we performed likelihood ratio tests comparing the models with fixed effects to the null models with only the random effects.

### Determination of gene expression by RT-qPCR.

Upon sacrifice of mice from gastrointestinal colonization experiments at 21 days postinoculation, cecal contents were mixed with RNAlater (Ambion, Life Technologies, Grand Island, NY) and frozen at −80°C. Samples were filtered through 250-µm polypropylene mesh (Small Parts, Inc., Logansport, IN) and then pelleted by centrifugation and resuspended in TRIzol (Life Technologies). RNA was extracted using mechanical disruption (bead beating with 0.5-mm-diameter zirconia-silica beads; BioSpec Products) and the PureLink kit TRIzol extraction procedure (Life Technologies) with on-column DNase I digestion. RNA concentration was determined with a NanoDrop ND-1000 spectrophotometer (Thermo Scientific). First-strand cDNA was synthesized using an oligo(dT) primer and SuperScript III reverse transcriptase (Life Technologies) and was subsequently diluted 10-fold with nuclease-free water; RNA samples were validated as DNA free via a no-reverse-transcription control. RT-qPCR mixtures (20 µl) contained diluted cDNA (2 µl), primers (0.2 µM; listed in Table 2; synthesized by IDT), and SYBR green PCR master mix (Applied Biosystems). Primer annealing temperature was optimized (between 59 and 63°C) on a StepOnePlus real-time PCR system (Applied Biosystems). Primer specificity was determined via melting curve analysis and agarose gel electrophoresis. Data were collected using a LightCycler 480 II (Roche), clear LightCycler 480 96-well plates (Roche), and the following cycling conditions (annealing and data acquisition temperatures shown in Table 2): 10 min at 95°C; 45 cycles of 95°C for 10 s, annealing for 30 s, and 1 s at data acquisition temperature; and melting curve (60 to 95°C, read every 0.3°C). PCR efficiency for each target was determined using a calibration curve. Quantification cycle (*C_q_*) values were exported from the LightCycler 480 instrument software using the second derivative maximum algorithm, and further analysis was performed in R (56). The suitability of six previously published reference genes (*ACT1*, *PMA1*, *RIP*, *RPP2B*, *LSC2*, and *TDH3* [59]) was tested as previously described (60) using SLqPCR (61); *ACT1*, *LSC2*, and *PMA1* were selected for use as reference genes (average expression stability *M* < 0.5; pairwise variability *V* = 0.16), and sample data were normalized to the geometric average for these genes. Normalized relative gene expression levels were log transformed, mean centered, and autoscaled as previously described (62). A one-way ANOVA was used to test for differences in expression between diets; *post hoc* pairwise *t* tests were performed using Bonferroni correction.

**TABLE 2:** RT-qPCR primers

Gene name	Assembly 19/21 identifier	Primer sequence		Temp (°C)	
		Forward primer	Reverse primer	Annealing	Reading
ACT1	orf19.5007	GTTGGTGATGAAGCCCAATC	CCCAGTTGGAAACAATACCG	62	72
CDC19	orf19.3575	TTGTCCACCAGTGGTCTTTC	AGCAGCTCTTTCGTTTCTGG	60	70
CTN1	orf19.4551	GGATTTGGTCCAGTTTCTGC	TGACGATGTTTGGAACTAGCAC	60	70
CTN3	orf19.2809	CTTTCCAGTGCCATGTCTTC	TGTTGCGGTAAGTGGCTATC	61	75
ICL1	orf19.6844	CAGCATTGGCTGTTGATGAC	TGGACAGTTTGACCGTAAGC	60	70
LSC2	orf19.1860	AGCTGAAGCCGGTAAATACG	GCCAAACCAGCACCATTAAC	60	73
MLS1	orf19.4833	CAGCTTTGGTTCCAGTTGTG	GTTCCTTTGGTGGCAATGAG	60	72
PMA1	orf19.5383	CCGAAGCCTTTGACAACTTC	CTCTTTGCATGGACACAAGG	60	73
POT1	orf19.7520	TGGCTGAAGCCAAAGGTTAC	GGACCAACACCCATGATTTC	61	75
POX1-3	orf19.1652	GAAGGCTCATCACTACCTGTTG	GCAATGGTGAGCAAGTATGG	60	72

### Fatty acid analysis.

Female Swiss Webster mice were fed diets containing coconut oil or beef tallow (30%), a diet containing both (12% coconut oil and 18% beef tallow), or the standard diet (AIN-93G) for 14 days and treated with antibiotics for 4 days as described above. Upon sacrifice, organ contents from throughout the GI tract (stomach; proximal, mid-, and distal small intestine; cecum; and colon) were harvested, flash-frozen, and stored at −80°C. For analysis, after addition of an internal standard (heptadecanoate), total lipids were extracted (63), followed by saponification and methylation (64). Fatty acid profiles were determined using an Autosystem XL gas chromatograph (PerkinElmer, Boston, MA) equipped with a 100-m by 0.25-mm-inside-diameter (i.d.) (film thickness, 0.25 µm) capillary column (SP-2560; Supelco) (65). Peaks of interest were identified by comparison with authentic fatty acid standards (Nu-Chek Prep, Inc., MN) and expressed as molar percent proportions of fatty acids relative to the internal standard or as micrograms of fatty acid per milligram (wet weight) of sample.

## References

[1] Gudlaugsson O, Gillespie S, Lee K, Vande Berg JV, Hu J, Messer S, Herwaldt L, Pfaller M, Diekema D 2003 Attributable mortality of nosocomial candidemia, revisited. Clin Infect Dis 37:1172–1177. doi:10.1086/378745. 14557960

[2] Diekema DJ, Pfaller MA 2004 Nosocomial candidemia: an ounce of prevention is better than a pound of cure. Infect Control Hosp Epidemiol 25:624–626. doi:10.1086/502451. 15357151

[3] Nucci M, Anaissie E 2001 Revisiting the source of candidemia: skin or gut? Clin Infect Dis 33:1959–1967. doi:10.1086/323759. 11702290

[4] Abi-Said D, Anaissie E, Uzun O, Raad I, Pinzcowski H, Vartivarian S 1997 The epidemiology of hematogenous candidiasis caused by different *Candida* species. Clin Infect Dis 24:1122–1128. doi:10.1086/513663. 9195068

[5] Aydemir C, Oguz SS, Dizdar EA, Akar M, Sarikabadayi YU, Saygan S, Erdeve O, Dilmen U 2011 Randomised controlled trial of prophylactic fluconazole versus nystatin for the prevention of fungal colonisation and invasive fungal infection in very low birth weight infants. Arch Dis Child Fetal Neonatal Ed 96:F164–F168. doi:10.1136/adc.2009.178996. 20659937

[6] Bertini G, Perugi S, Dani C, Filippi L, Pratesi S, Rubaltelli FF 2005 Fluconazole prophylaxis prevents invasive fungal infection in high-risk, very low birth weight infants. J Pediatr 147:162–165. doi:10.1016/j.jpeds.2005.02.020. 16126042

[7] Clerihew L, Austin N, McGuire W 2007 Prophylactic systemic antifungal agents to prevent mortality and morbidity in very low birth weight infants. Cochrane Database Syst Rev 4:CD003850. doi:10.1002/14651858.CD003850.pub3:CD003850. 17943803

[8] Faiz S, Neale B, Rios E, Campos T, Parsley E, Patel B, Ostrosky-Zeichner L 2009 Risk-based fluconazole prophylaxis of *Candida* bloodstream infection in a medical intensive care unit. Eur J Clin Microbiol Infect Dis 28:689–692. doi:10.1007/s10096-008-0666-4. 19011913

[9] Manzoni P, Arisio R, Mostert M, Leonessa M, Farina D, Latino MA, Gomirato G 2006 Prophylactic fluconazole is effective in preventing fungal colonization and fungal systemic infections in preterm neonates: a single-center, 6-year, retrospective cohort study. Pediatrics 117:e22–e32. doi:10.1542/peds.2004-2227. 16326690

[10] Manzoni P, Stolfi I, Pugni L, Decembrino L, Magnani C, Vetrano G, Tridapalli E, Corona G, Giovannozzi C, Farina D, Arisio R, Merletti F, Maule M, Mosca F, Pedicino R, Stronati M, Mostert M, Gomirato G, Italian Task Force for the Study and Prevention of Neonatal Fungal Infections, Italian Society of Neonatology 2007 A multicenter, randomized trial of prophylactic fluconazole in preterm neonates. N Engl J Med 356:2483–2495. doi:10.1056/NEJMoa065733. 17568029

[11] McCrossan BA, McHenry E, O’Neill F, Ong G, Sweet DG 2007 Selective fluconazole prophylaxis in high-risk babies to reduce invasive fungal infection. Arch Dis Child Fetal Neonatal Ed 92:F454–F458. doi:10.1136/adc.2006.094359. 17460023 PMC2675390

[12] Rolnitsky A, Levy I, Sirota L, Shalit I, Klinger G 2012 Targeted fluconazole prophylaxis for high-risk very low birth weight infants. Eur J Pediatr 171:1481–1487. doi:10.1007/s00431-012-1760-2. 22628137

[13] Uko S, Soghier LM, Vega M, Marsh J, Reinersman GT, Herring L, Dave VA, Nafday S, Brion LP 2006 Targeted short-term fluconazole prophylaxis among very low birth weight and extremely low birth weight infants. Pediatrics 117:1243–1252. doi:10.1542/peds.2005-1969. 16585321

[14] Weitkamp J, Ozdas A, LaFleur B, Potts AL 2008 Fluconazole prophylaxis for prevention of invasive fungal infections in targeted highest risk preterm infants limits drug exposure. J Perinatol 28:405–411. doi:10.1038/sj.jp.7211914. 18185518

[15] Healy CM, Campbell JR, Zaccaria E, Baker CJ 2008 Fluconazole prophylaxis in extremely low birth weight neonates reduces invasive candidiasis mortality rates without emergence of fluconazole-resistant *Candida* species. Pediatrics 121:703–710. doi:10.1542/peds.2007-1130. 18381534

[16] Kaufman D 2008 Fluconazole prophylaxis decreases the combined outcome of invasive *Candida* infections or mortality in preterm infants. Pediatrics 122:1158–1159. doi:10.1542/peds.2008-1837. 18978001

[17] Playford EG, Webster AC, Sorrell TC, Craig JC 2006 Antifungal agents for preventing fungal infections in non-neutropenic critically ill and surgical patients: systematic review and meta-analysis of randomized clinical trials. J Antimicrob Chemother 57:628–638. doi:10.1093/jac/dki491. 16459344

[18] Vardakas KZ, Samonis G, Michalopoulos A, Soteriades ES, Falagas ME 2006 Antifungal prophylaxis with azoles in high-risk, surgical intensive care unit patients: a meta-analysis of randomized, placebo-controlled trials. Crit Care Med 34:1216–1224. doi:10.1097/01.CCM.0000208357.05675.C3. 16484923

[19] Anderson JB 2005 Evolution of antifungal-drug resistance: mechanisms and pathogen fitness. Nat Rev Microbiol 3:547–556. doi:10.1038/nrmicro1179. 15953931

[20] Cowen LE, Anderson JB, Kohn LM 2002 Evolution of drug resistance in *Candida albicans*. Annu Rev Microbiol 56:139–165. doi:10.1146/annurev.micro.56.012302.160907. 12142485

[21] Rex JH 2006 Antifungal prophylaxis in the intensive care unit: who should get it? Crit Care Med 34:1286–1287. doi:10.1097/01.CCM.0000208110.36504.19. 16550099

[22] Sardi JCO, Scorzoni L, Bernardi T, Fusco-Almeida AM, Mendes Giannini MJ 2013 *Candida* species: current epidemiology, pathogenicity, biofilm formation, natural antifungal products and new therapeutic options. J Med Microbiol 62:10–24. doi:10.1099/jmm.0.045054-0. 23180477

[23] Centers for Disease Control and Prevention 2013 Antibiotic resistance threats in the United States, 2013. Centers for Disease Control and Prevention, Atlanta, GA http://www.cdc.gov/drugresistance/threat-report-2013/.

[24] De Wit N, Derrien M, Bosch-Vermeulen H, Oosterink E, Keshtkar S, Duval C, de Vogel-van den Bosch J, Kleerebezem M, Muller M, van der Meer R 2012 Saturated fat stimulates obesity and hepatic steatosis and affects gut microbiota composition by an enhanced overflow of dietary fat to the distal intestine. Am J Physiol Gastrointest Liver Physiol 303:G589–G599. doi:10.1152/ajpgi.00488.2011. 22700822

[25] Hildebrandt MA, Hoffmann C, Sherrill-Mix SA, Keilbaugh SA, Hamady M, Chen Y, Knight R, Ahima RS, Bushman F, Wu GD 2009 High-fat diet determines the composition of the murine gut microbiome independently of obesity. Gastroenterology 137:1716–1724. doi:10.1053/j.gastro.2009.08.042. 19706296 PMC2770164

[26] Murphy EF, Cotter PD, Healy S, Marques TM, O’Sullivan O, Fouhy F, Clarke SF, O’Toole PW, Quigley EM, Stanton C, Ross PR, O’Doherty RM, Shanahan F 2010 Composition and energy harvesting capacity of the gut microbiota: relationship to diet, obesity and time in mouse models. Gut 59:1635–1642. doi:10.1136/gut.2010.215665. 20926643

[27] Turnbaugh PJ, Bäckhed F, Fulton L, Gordon JI 2008 Diet-induced obesity is linked to marked but reversible alterations in the mouse distal gut microbiome. Cell Host Microbe 3:213–223. doi:10.1016/j.chom.2008.02.015. 18407065 PMC3687783

[28] Turnbaugh PJ, Ridaura VK, Faith JJ, Rey FE, Knight R, Gordon JI 2009 The effect of diet on the human gut microbiome: a metagenomic analysis in humanized gnotobiotic mice. Sci Transl Med 1:6ra14. doi:10.1126/scitranslmed.3000322. PMC2894525 20368178

[29] Maukonen J, Saarela M 2015 Human gut microbiota: does diet matter? Proc Nutr Soc 74:23–36. doi:10.1017/S0029665114000688. 25156389

[30] Shen W, Gaskins HR, McIntosh MK 2014 Influence of dietary fat on intestinal microbes, inflammation, barrier function and metabolic outcomes. J Nutr Biochem 25:270–280. doi:10.1016/j.jnutbio.2013.09.009. 24355793

[31] Ogbolu DO, Oni AA, Daini OA, Oloko AP 2007 *In vitro* antimicrobial properties of coconut oil on *Candida* species in Ibadan, Nigeria. J Med Food 10:384–387. doi:10.1089/jmf.2006.1209. 17651080

[32] Kabara JJ, Swieczkowski DM, Conley AJ, Truant JP 1972 Fatty acids and derivatives as antimicrobial agents. Antimicrob Agents Chemother 2:23–28. doi:10.1128/AAC.2.1.23. 4670656 PMC444260

[33] Bergsson G, Arnfinnsson J, Steingrimsson O, Thormar H 2001 *In vitro* killing of *Candida albicans* by fatty acids and monoglycerides. Antimicrob Agents Chemother 45:3209–3212. doi:10.1128/AAC.45.11.3209-3212.2001. 11600381 PMC90807

[34] Strijbis K, van Roermund CWT, Visser WF, Mol EC, van den Burg J, MacCallum DM, Odds FC, Paramonova E, Krom BP, Distel B 2008 Carnitine-dependent transport of acetyl coenzyme A in *Candida albicans* is essential for growth on nonfermentable carbon sources and contributes to biofilm formation. Eukaryot Cell 7:610–618. doi:10.1128/EC.00017-08. 18281597 PMC2292619

[35] Zhou H, Lorenz MC 2008 Carnitine acetyltransferases are required for growth on non-fermentable carbon sources but not for pathogenesis in *Candida albicans*. Microbiology 154:500–509. doi:10.1099/mic.0.2007/014555-0. 18227254

[36] Carman AJ, Vylkova S, Lorenz MC 2008 Role of acetyl coenzyme A synthesis and breakdown in alternative carbon source utilization in *Candida albicans*. Eukaryot Cell 7:1733–1741. doi:10.1128/EC.00253-08. 18689527 PMC2568070

[37] Prigneau O, Porta A, Maresca B 2004 *Candida albicans* CTN gene family is induced during macrophage infection: homology, disruption and phenotypic analysis of CTN3 gene. Fungal Genet Biol 41:783–793. doi:10.1016/j.fgb.2004.04.001. 15219562

[38] Ramirez MA, Lorenz MC 2009 The transcription factor homolog CTF1 regulates β-oxidation in *Candida albicans*. Eukaryot Cell 8:1604–1614. doi:10.1128/EC.00206-09. 19700635 PMC2756860

[39] Strijbis K, Distel B 2010 Intracellular acetyl unit transport in fungal carbon metabolism. Eukaryot Cell 9:1809–1815. doi:10.1128/EC.00172-10. 20889721 PMC3008284

[40] Kunau W, Dommes V, Schulz H 1995 Beta-oxidation of fatty acids in mitochondria, peroxisomes, and bacteria: a century of continued progress. Prog Lipid Res 34:267–342. doi:10.1016/0163-7827(95)00011-9. 8685242

[41] Agricultural Research Service 2015 Full report (all nutrients) 04047, oil, coconut. Agricultural Research Service, US Department of Agriculture, Washington, DC.

[42] Barelle CJ, Priest CL, Maccallum DM, Gow NAR, Odds FC, Brown AJP 2006 Niche-specific regulation of central metabolic pathways in a fungal pathogen. Cell Microbiol 8:961–971. doi:10.1111/j.1462-5822.2005.00676.x. 16681837 PMC1472618

[43] Piekarska K, Mol E, van den Berg M, Hardy G, van den Burg J, van Roermund C, MacCallum D, Odds F, Distel B 2006 Peroxisomal fatty acid beta-oxidation is not essential for virulence of *Candida albicans*. Eukaryot Cell 5:1847–1856. doi:10.1128/EC.00093-06. 16963628 PMC1694795

[44] Prigneau O, Porta A, Poudrier JA, Colonna-Romano S, Noël T, Maresca B 2003 Genes involved in beta-oxidation, energy metabolism and glyoxylate cycle are induced by *Candida albicans* during macrophage infection. Yeast 20:723–730. doi:10.1002/yea.998. 12794933

[45] Ramirez MA, Lorenz MC 2007 Mutations in alternative carbon utilization pathways in *Candida albicans* attenuate virulence and confer pleiotropic phenotypes. Eukaryot Cell 6:280–290. doi:10.1128/EC.00372-06. 17158734 PMC1797957

[46] Lorenz MC, Fink GR 2001 The glyoxylate cycle is required for fungal virulence. Nature 412:83–86. doi:10.1038/35083594. 11452311

[47] Brown AJP, Brown GD, Netea MG, Gow NAR 2014 Metabolism impacts upon Candida immunogenicity and pathogenicity at multiple levels. Trends Microbiol 22:614–622. doi:10.1016/j.tim.2014.07.001. 25088819 PMC4222764

[48] Fradin C, De Groot P, MacCallum D, Schaller M, Klis F, Odds FC, Hube B 2005 Granulocytes govern the transcriptional response, morphology and proliferation of Candida albicans in human blood. Mol Microbiol 56:397–415. doi:10.1111/j.1365-2958.2005.04557.x. 15813733

[49] Lorenz MC, Bender JA, Fink GR 2004 Transcriptional response of *Candida albicans* upon internalization by macrophages. Eukaryot Cell 3:1076–1087. doi:10.1128/EC.3.5.1076-1087.2004. 15470236 PMC522606

[50] Marten B, Pfeuffer M, Schrezenmeir J 2006 Medium-chain triglycerides. Int Dairy J 16:1374–1382. doi:10.1016/j.idairyj.2006.06.015.

[51] Agricultural Research Service 2012 Nutrient intakes from food: mean amounts consumed per individual, by gender and age; what we eat in America, NHANES 2009–2010. Agricultural Research Service, US Department of Agriculture, Washington, DC http://www.ars.usda.gov/ba/bhnrc/fsrg.

[52] Vargas SL, Patrick CC, Ayers GD, Hughes WT 1993 Modulating effect of dietary carbohydrate supplementation on *Candida albicans* colonization and invasion in a neutropenic mouse model. Infect Immun 61:619–626. 8423091 10.1128/iai.61.2.619-626.1993 PMC302772

[53] Weig M, Werner E, Frosch M, Kasper H 1999 Limited effect of refined carbohydrate dietary supplementation on colonization of the gastrointestinal tract of healthy subjects by *Candida albicans*. Am J Clin Nutr 69:1170–1173. 10357735 10.1093/ajcn/69.6.1170

[54] Reeves PG, Nielsen FH, Fahey GC Jr. 1993 AIN-93 purified diets for laboratory rodents: final report of the American Institute of Nutrition ad hoc writing committee on the reformulation of the AIN-76A rodent diet. J Nutr 123:1939–1951. 8229312 10.1093/jn/123.11.1939

[55] White SJ, Rosenbach A, Lephart P, Nguyen D, Benjamin A, Tzipori S, Whiteway M, Mecsas J, Kumamoto CA 2007 Self-regulation of *Candida albicans* population size during GI colonization. PLoS Pathog 3:e184. doi:10.1371/journal.ppat.0030184. 18069889 PMC2134954

[56] R Core Team 2012 R: a language and environment for statistical computing. R Foundation for Statistical Computing, Vienna, Austria.

[57] Pinheiro J, Bates D, DebRoy S, Sarkar D, R Core Team 2015 nlme: linear and nonlinear mixed effects models. R package version 3.1-122 R Foundation for Statistical Computing, Vienna, Austria.

[58] Hothorn T, Bretz F, Westfall P 2008 Simultaneous inference in general parametric models. Biom J 50:346–363. doi:10.1002/bimj.200810425. 18481363

[59] Nailis H, Coenye T, Van Nieuwerburgh F, Deforce D, Nelis HJ 2006 Development and evaluation of different normalization strategies for gene expression studies in *Candida albicans* biofilms by real-time PCR. BMC Mol Biol 7:25. doi:10.1186/1471-2199-7-25. 16889665 PMC1557526

[60] Vandesompele J, De Preter K, Pattyn F, Poppe B, Van Roy N, De Paepe A, Speleman F 2002 Accurate normalization of real-time quantitative RT-PCR data by geometric averaging of multiple internal control genes. Genome Biol 3:RESEARCH0034. doi:10.1186/gb-2002-3-7-research0034. 12184808 PMC126239

[61] Kohl M 2007 SLqPCR: functions for analysis of real-time quantitative PCR data at SIRS-Lab GmbH. SIRS-Lab GmbH, Jena, Germany.

[62] Willems E, Leyns L, Vandesompele J 2008 Standardization of real-time PCR gene expression data from independent biological replicates. Anal Biochem 379:127–129. doi:10.1016/j.ab.2008.04.036. 18485881

[63] Folch J, Lees M, Sloane SGH 1957 A simple method for the isolation and purification of total lipides from animal tissues. J Biol Chem 226:497–509. 13428781

[64] Morrison WR, Smith LM 1964 Preparation of fatty acid methyl esters and dimethylacetals from lipids with boron-fluoride–methanol. J Lipid Res 5:600–608. 14221106

[65] Matthan NR, Ip B, Resteghini N, Ausman LM, Lichtenstein AH 2010 Long-term fatty acid stability in human serum cholesteryl ester, triglyceride, and phospholipid fractions. J Lipid Res 51:2826–2832. doi:10.1194/jlr.D007534. 20448292 PMC2918465

